# Effects of combined extract of cocoa, coffee, green tea and garcinia on lipid profiles, glycaemic markers and inflammatory responses in hamsters

**DOI:** 10.1186/s12906-015-0806-1

**Published:** 2015-08-12

**Authors:** Chih-Wei Chang, Yi-Ju Hsu, Yi-Ming Chen, Wen-Ching Huang, Chi-Chang Huang, Mei-Chich Hsu

**Affiliations:** Department of Sports Medicine, Kaohsiung Medical University, 100, Shih-Chuan 1st Rd, Sanmin, Kaohsiung 80708 Taiwan; Graduate Institute of Sports Science, National Taiwan Sport University, 250, Wen-Hua 1st Rd, Guishan, Taoyuan 33301 Taiwan; Graduate Institute of Athletics and Coaching Science, National Taiwan Sport University, 250, Wen-Hua 1st Rd, Taoyuan, 33301 Taiwan

**Keywords:** Cholesterol, Dyslipidaemia, Polyphenols, Plant, Dose-dependent, HOMA-IR

## Abstract

**Background:**

Dyslipidaemia is highly associated with cardiovascular and cerebrovascular diseases, which have been ranked second and third place of leading causes of death in Taiwan. Some plant extracts have been proved effective against dyslipidaemia. However, the combination of plant extracts was rarely studied. The purpose of the present study is to understand the beneficial effects of a combined extract (comprising cocoa, coffee, green tea and garcinia; CCGG) on lipid profiles in serum, liver, and faeces as well as glycaemic markers and related proinflammatory cytokines by using an appropriate animal model, the golden Syrian hamster.

**Methods:**

A total of 40 male hamsters were randomly assigned to five groups: (1) vehicle control, (2) high-cholesterol diet control, (3) high-cholesterol diet of 311 mg/kg/d of CCGG, (4) high-cholesterol diet of 622 mg/kg/d of CCGG and (5) high-cholesterol diet of 1555 mg/kg/d of CCGG. At the end of the experiment, blood, tissue and faecal samples were collected for further analysis.

**Results:**

After 6 weeks of treatment, CCGG supplementation significantly reduced serum lipid content (triglycerides, total cholesterol and LDL-C) and hepatic lipid content (triglycerides and cholesterol) with dose-dependent effects. In addition, an increase in excretion of faecal lipids (bile acids) was observed after supplementation. Furthermore, the homeostasis model assessment of insulin resistance (HOMA-IR) index and serum proinflammatory cytokine levels (TNF-α and IL-6) involved in dyslipidaemia was markedly improved. In addition, by monitoring biochemical parameters as well as histopathology of major tissues, no toxicity was observed after the consumption of CCGG.

**Conclusion:**

Dietary CCGG supplementation may exert potential effects on ameliorating hyperlipidaemia, insulin resistance, liver steatosis and related inflammation.

**Electronic supplementary material:**

The online version of this article (doi:10.1186/s12906-015-0806-1) contains supplementary material, which is available to authorized users.

## Background

As time has progressed and society advances, indulgent lifestyles have produced “lifestyle diseases”, particularly dyslipidaemia. Dyslipidaemia is a disease characterised by an abnormal amount of lipids in the blood, which can result in a predisposition to coronary, cerebrovascular and peripheral vascular arterial diseases. In addition, systemic inflammation has been proposed to be accompanied by dyslipidaemia, producing several proinflammatory factors (including tumour necrosis factor (TNF)-α and interleukin 6 (IL-6)) [1, 2]. Since 2000, cardiovascular and cerebrovascular diseases have been ranked second and third place of leading causes of death in Taiwan. According to the Industry & Technology Intelligence Service (Taiwan), dyslipidaemia prevalence has exceeded 10 % of the population (older than 15 y) in Taiwan. Moreover, dyslipidaemia is observed in the younger population. Dyslipidaemia severely threatens health and is associated with exorbitant medical expenses.

Current blood lipid-lowering drugs have a higher efficacy and potency than before; however, some adverse effects still occur when medication is taken. Minor gastrointestinal complaints including nausea, cramping and constipation occur when bile acid resins are taken [3]. Niacin appears to cause cutaneous flushing and itching [3]. Statins and fibric acids may be associated with increased serum aminotransferase levels, creatine kinase levels, myopathy and rhabdomyolysis [3]. Diet control and increased physical activity are frequently encouraged to ameliorate dyslipidaemia [3]. Studies [4–7] have revealed that consuming plant or natural extract supplements is beneficial. Plant polyphenols have recently gained increasing attention because of their potent properties against blood lipids and related inflammatory responses. Cocoa, coffee, green tea and garcinia (CCGG) contain abundant polyphenols, particularly proanthocyanidins, chlorogenic acids, catechins and xanthones, respectively [8–11]. Polyphenols potentially reduce blood lipids and have an antiinflammatory effect [12–17].

Regarding to CCGG, there were some obvious evidences for CCGG against dyslipidaemia stated in rodent and *in vitro* studies. Nwichi et al. [18] demonstrated an 8-weeks administration of cocoa extract exhibited hypolipidaemic effects in cholesterol-fed rats. Song et al. [19] demonstrated that an 11-week consumption of decaffeinated green coffee bean significantly reduced plasma lipids in high-fat diet fed mice. Yan et al. [20] studied the 6-weeks effect of green tea polysaccharides and polyphenols in high-fat diet fed rats, both of them showed effective reduction in serum lipids. Bumrungpert et al. [21] revealed the effects of mangosteen against inflammation and insulin resistance in human adipocytes. Despite of the fact that previous studies have stated the beneficial effects of diverse polyphenol-rich extracts in isolation, very few studies have examined the combination. However, combined ingredients may enhance the benefit and reduce the effective dose [22], also exert a synergistic effect [23].

Since the early 1980s, an animal model, the golden Syrian hamster, has been employed to assess diet-induced atherosclerosis [24]. In general, using unmodified rats or mice as animal models is unsuitable for examining diet-induced changes in blood lipids because they do not develop aortic lesions or an atherogenic lipoprotein profile (nHDL-C > HDL-C) similar to human beings [25]. Hamsters are a more appropriate animal model. Recently, numerous studies have adopted hamsters to investigate the effect of plant extracts on blood lipids [5, 26, 27]. Therefore, in the present study, we examined the potential effects and dose-response relationship of combined extract (composed of CCGG) on lipid profiles, glycaemic markers and inflammatory responses in hamsters. We monitored common toxicity markers that may be influenced after the intake of CCGG.

## Methods

### Diets and chemicals

A commercially available supplement consisting of CCGG (beans of *Theobroma cacao*, beans of *Coffea robusta*, leaves of *Camellia sinensis* and fruits of *Garcinia mangostana*) was provided by Sunrider International (CA, USA) and employed as dietary treatment. The nutritional information of the supplement is shown in Table 1. To determine the total polyphenolic content, CCGG was extracted using a solvent in a 4:6 ratio of methanol and water (v/v) with 0.5 % hydrochloric acid. Aliquots of a 50-μL solution containing extract or a standard solution of gallic acid were mixed with 1 mL of a 2 % w/v sodium carbonate solution and allowed to stand for 2 min with intermittent shaking. Subsequently, 50 μL of 50 % v/v Folin–Ciocalteu reagent (Sigma-Aldrich, St. Louis, MO, USA) was added and incubated for 10 min in the dark. Absorbance was spectrophotometrically read at 750 nm. To determine epigallocatechin gallate (EGCG) and α-mangostin, a high-performance liquid chromatograph (HPLC; Hitachi, Tokyo, Japan) equipped with a LiChroCART 250-4,6 column, ultraviolet-visible spectroscopy L-2420 detector, L-2200 autosampler and L-2130 pump was used. The HPLC conditions for analysing EGCG and α-mangostin were slightly modified from those described in previous studies [28, 29]. All of the analyses were performed adequately in three replicates. The total polyphenol, EGCG and α-mangostin contents in CCGG were 196 mg/g, 4.0 mg/g and 1.5 mg/g, respectively.

**Table 1:** The nutritional information of CCGG

Energy content	5.2 kcal/g
Content (mg/g)	
Protein	30
Total fat	290
Saturated fat	4
Trans fat	0
Carbohydrate	610
Sodium	0.05
Dietary fiber	50
Iron	0.003
Calcium	0.02

An animal diet was LabDiet 5001 rodent diet (LabDiet, St. Louis, MO, USA) in a vehicle control group (vehicle), and an additional 0.2 % cholesterol and 10 % lard were added to the diet for a high-cholesterol diet group (HCD).

### Animals and treatment

The animal use protocol was submitted to and had been approved by the Institutional Animal Care and Use Committee (IACUC) of National Taiwan Sport University (Taoyuan, Taiwan). The IACUC approval number of the present study was IACUC-10210 and was valid from 10/20/2013 to 12/31/2014. We declared all animal experiments were conducted according to the protocol. Male golden Syrian hamsters (4 weeks old) were purchased from the National Laboratory Animal Center (Taipei, Taiwan) and maintained in 12 h of light/dark at a temperature of 24 ± 2 °C and 65 ± 5 % humidity with access to food and water ad libitum. The hamsters were monitored daily for food intake. Body weight and water intake were recorded two times per week. The food efficiency was calculated at the end of the experiments. After 4 weeks of acclimatisation, the hamsters were randomised into five groups (n = 8 per group): (1) vehicle control (Vehicle), (2) high-cholesterol diet control (HCD), (3) high-cholesterol diet with 311 mg/kg/d of CCGG (CCGG-1X), (4) high-cholesterol diet with 622 mg/kg/d of CCGG (CCGG-2X) and (5) high-cholesterol diet with 1555 mg/kg/d of CCGG (CCGG-5X). The hamster CCGG dose (311 mg/kg/d) was converted from a human equivalent dose (HED) based on body surface area by the following formula from the US Food and Drug Administration: assuming a human weight of 60 kg, the HED for 2.5 (g/d) ÷ 60 (kg) = 0.042 × 7.4 = hamster dose of 311 mg/kg/d. The conversion coefficient 7.4 was used to account for differences in body surface area between a hamster and a human. CCGG was dissolved using deionised water and administered to the hamsters by gastric tube in a dose of 0.01 mL/g. At the end of week 6, the hamsters were anaesthetised with 5 % isoflurane at the rate of 0.5 L/min and euthanised by exsanguination after 12 h of food deprivation. Blood samples were collected from the abdominal aortas. The hearts, livers, kidneys, lungs and fat depots (epididymal fat) were collected and weighed. Serum samples were isolated using centrifugation at 2000 × g for 15 min. All of the samples were snap-frozen and stored at -80 °C until further analysis.

### Biochemical analysis of serum samples

The biochemical parameters, including aspartate aminotransferase (AST), alanine transaminase (ALT), alkaline phosphatase (Alk-P), lactate dehydrogenase (LDH), creatine phosphokinase (CPK), albumin, total bilirubin, total protein (TP), blood urea nitrogen (BUN), creatinine, uric acid (UA) and glucose, were analysed. Blood lipid parameters, including total cholesterol (TC), triglycerides (TG), low-density lipoprotein cholesterol (LDL-C) and high-density lipoprotein cholesterol (HDL-C), were measured using an autoanalyser (Hitachi, Tokyo, Japan). An adiponectin ELISA kit (Assaypro, St. Charles, MO, USA), insulin ELISA kit (BlueGene, Shanghai, China), TNF-α ELISA kit (Randox, Antrim, UK) and IL-6 ELISA kit (Cusabio, Hubei, China) were used according to the manufacturer’s protocols to determine the serum levels of adiponectin, insulin, TNF-α, and IL-6. The homeostasis model assessment of insulin resistance (HOMA-IR) was then calculated according to the homeostasis of the assessment by using the following equation [30]:

$$ \mathrm{HOMA}-\mathrm{I}\mathrm{R} = \left[\mathrm{glucose}\ \left(\mathrm{mmol}/\mathrm{L}\right) \times \mathrm{insulin}\ \left(\upmu \mathrm{L}\ \mathrm{U}/\mathrm{mL}\right)\right]\ /\ 22.51 $$

### Liver lipid content

To determine liver cholesterol and triglycerides, 20 mg of liver tissue was homogenised in a 200-μL solvent (chloroform:isopropanol:NP40 = 7:11:0.1). Centrifuged at 12 000 × g for 10 min, an aliquot of 100 μL was extracted and dried. The pellet was reconstituted with a buffer (1 M of potassium phosphate, pH = 7.4, 500 mM of sodium chloride, 50 mM of cholic acid), and water bath sonication was employed to dissolve the precipitate. A Cholesterol Fluorometric Assay Kit (Cayman, Ann Arbor, MI, USA) and Triglyceride Colorimetric Assay Kit (Cayman, Ann Arbor, MI, USA) were used to analyse liver cholesterol and triglyceride contents.

### Faecal lipid content

Faeces were collected and dried in an oven at Week 6. Subsequently, 0.1 g of faeces were pulverised in 1 mL of phosphate buffered saline and extracted using a solvent (chloroform:methanol = 2:1). The organic phase was filtered using filter paper (Whatman NO.5). Afterwards, the residue was dried and reconstituted with 1 mL of dimethyl sulphoxide. Water bath sonication was used to dissolve the precipitate. A cholesterol liquid assay (Randox, Antrim, UK), Triglyceride Colorimetric Assay Kit (Cayman, Ann Arbor, MI, USA) and Total Bile Acids Assay Kit (Crystal Chem, Downers Grove, IL, USA) were adopted to analyse faecal cholesterol, triglyceride and bile acid contents.

### Histopathology of tissues

Liver, fat, heart, kidney and lung tissues were removed at the end of the experiment, fixed in 10 % buffered formalin, and then embedded in paraffin. The paraffin-embedded samples were sectioned and underwent haematoxylin and eosin (H&E) staining under a light microscope equipped with a charge-coupled device camera (Olympus, Tokyo, Japan) by a clinical pathologist.

### Statistical analysis

Data are expressed as mean ± standard error of the mean (SEM). One-way ANOVA with Tukey’s posttest and Pearson correlation for dose-dependent effect were used for all data comparisons. A P < 0.05 was statistically significant. Statistical analyses were conducted using the SPSS 19.0.

## Results

### Effects of CCGG supplementation on body weight, organ weight, and diet intake

**Fig. 1:**
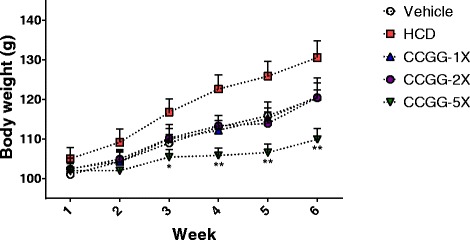
Body weights over the course of 6 weeks. Values are expressed as mean ± SEM. Body weights of each group were compared by one-way ANOVA with Tukey’s posttest. (* *P* < 0.05, ** *P* < 0.01 compared with HCD). Vehicle; vehicle control, HCD; high-cholesterol diet control, CCGG-1X; high-cholesterol diet with 311 mg/kg/d of CCGG, CCGG-2X; high-cholesterol diet with 622 mg/kg/d of CCGG, CCGG-5X; high-cholesterol diet with 1555 mg/kg/d of CCGG

**Fig. 2:**
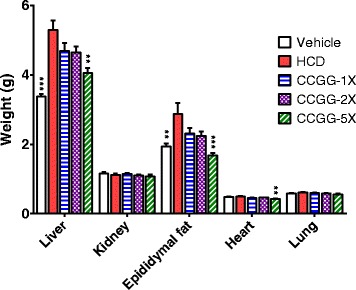
Tissue weights at the end of the experiment. Values are expressed as mean ± SEM. Statistical significance was determined using the one-way ANOVA with Tukey’s posttest. **P < 0.01, ***P < 0.001 compared with HCD. Vehicle; vehicle control, HCD; high-cholesterol diet control, CCGG-1X; high-cholesterol diet with 311 mg/kg/d of CCGG, CCGG-2X; high-cholesterol diet with 622 mg/kg/d of CCGG, CCGG-5X; high-cholesterol diet with 1555 mg/kg/d of CCGG

After CCGG treatment in the hamsters, significant differences in body weight were observed between the CCGG-5X and HCD groups from Week 3 (Fig. 1). The body weight of the CCGG-1X and CCGG-2X groups were slightly nonsignificantly lower than that of the HCD group. The average final body weight of the CCGG-5X group decreased significantly by 15.9 % (P < 0.01) compared with the HCD group without affecting food and water intake (Table. 2). Furthermore, the CCGG-supplementation yielded a low food efficiency ratio. In addition, tissue weights were measured at the end of the study (Fig. 2). The weights of the liver, epididymal fat and heart tissues decreased significantly in the CCGG-5X group (by 23.4 %, 41.4 % and 14.4 %, respectively) compared with the HCD group. No significant change was observed in the weight of the kidney and lung among the groups (P > 0.05 vs. the HCD group).

**Table 2:** Final body weight and effects of CCGG supplementation on food intake, water intake and food efficiency ratio

	Vehicle		HCD		CCGG-1X		CCGG-2X		CCGG-5X	
	Mean	SEM	Mean	SEM	Mean	SEM	Mean	SEM	Mean	SEM
Final body weight (g/hamster)	120.4	2.0	130.6	4.2	120.7	3.7	120.5	3.7	109.9**	2.8
Food intake (g/hamster/week)	71.8	6.9	68.5	6.0	66.0	6.5	76.1	9.6	80.6	9.0
Water intake (g/hamster/week)	78.8**	4.2	60.6	3.8	62.1	3.0	59.4	3.3	51.6	2.7
Food efficiency ratio (%)	4.2	0.8	5.4	0.9	4.7	0.9	3.9	1.1	1.2*	0.6

Values are expressed as mean ± SEM. Statistical significance was determined using the one-way ANOVA with Tukey’s posttest

*P < 0.05, **P < 0.01 compared with HCD

Food efficiency ratio = Body weight gain (g)/Food intake (g)

Vehicle; vehicle control, HCD; high-cholesterol diet control, CCGG-1X; high-cholesterol diet with 311 mg/kg/d of CCGG, CCGG-2X; high-cholesterol diet with 622 mg/kg/d of CCGG, CCGG-5X; high-cholesterol diet with 1555 mg/kg/d of CCGG

### Effects of CCGG supplementation on serum lipid profiles

**Fig. 3:**
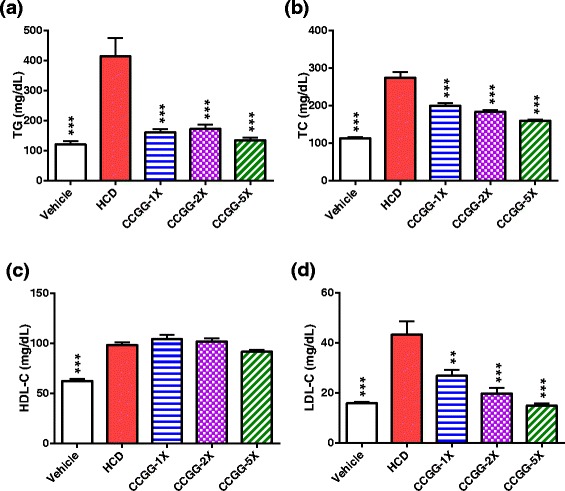
Effects of CCGG supplementation on serum lipids. **a**; serum triglycerides levels. **b**; serum total cholesterol levels. **c**; serum high-density lipoprotein cholesterol. **d**; serum low-density lipoprotein cholesterol. Values are expressed as mean ± SEM. Statistical significance was determined using the one-way ANOVA with Tukey’s posttest. **P < 0.01, ***P < 0.001 compared with HCD. Vehicle; vehicle control, HCD; high-cholesterol diet control, CCGG-1X; high-cholesterol diet with 311 mg/kg/d of CCGG, CCGG-2X; high-cholesterol diet with 622 mg/kg/d of CCGG, CCGG-5X; high-cholesterol diet with 1555 mg/kg/d of CCGG

An HCD diet fed to the hamsters significantly increased the serum levels of TG, TC, HDL-C and LDL-C by 245.6 %, 142.6 %, 57.5 % and 174.1 %, respectively (P < 0.001 vs. the vehicle group) (Fig. 3). After the CCGG supplementation, the treated groups exhibited a significant decrease in the serum levels of TG (61.3 %, 58.7 % and 67.6 % reduction in the CCGG-1X, CCGG-2X and CCGG-5X groups, respectively), TC (27.4 %, 33.3 % and 41.8 % reduction in the CCGG-1X, CCGG-2X and CCGG-5X groups, respectively), and LDL-C (38.0 %, 54.3 % and 65.7 % reduction in the CCGG-1X, CCGG-2X and CCGG-5X groups, respectively) compared with the HCD group. Strongly dose-dependent effects were observed in the serum levels of TC (r = -0.729, P < 0.001) and LDL-C (r = -0.676, P < 0.001). No significant changes were found in the HDL-C between the HCD group and treated groups. Furthermore, no significant differences in serum adiponectin levels were observed among the groups (Table 3).

**Table 3:** Effects of CCGG supplementation on serum biochemical analysis

	Vehicle		HCD		CCGG-1X		CCGG-2X		CCGG-5X	
	Mean	SEM	Mean	SEM	Mean	SEM	Mean	SEM	Mean	SEM
Adiponectin (ng/mL)	33.17	0.74	31.36	0.74	31.65	1.57	36.61	2.70	36.38	1.29
Glucose (mmol/L)	8.64	0.56	10.25	0.53	8.53	0.30	8.19	0.52	8.17	0.58
Albumin (g/dL)	3.40	0.05	3.34	0.06	3.33	0.05	3.23	0.05	3.19	0.05
AST (U/L)	56.5	3.2	46.4	1.8	56.9	3.0	47.1	2.9	45.3	2.6
ALT (U/L)	68.1	2.7	88.3	4.1	82.5	4.9	101.1	10.9	93.8	5.2
Alk-P (U/L)	112.1	3.8	103.3	3.6	115.0	4.0	115.6	3.4	116.4	7.8
Bilirubin (mg/dL)	0.38	0.03	0.34	0.03	0.33	0.04	0.35	0.02	0.35	0.03
TP (g/dL)	5.55	0.08	5.55	0.10	5.48	0.05	5.35	0.10	5.26	0.09
UA (mg/dL)	0.98	0.06	0.85	0.07	1.22	0.18	1.19	0.13	1.15	0.11
Creatinine (mg/dL)	0.18	0.02	0.14	0.01	0.16	0.01	0.13	0.01	0.17	0.01
BUN (mg/dL)	20.21**	0.67	15.89	0.94	19.17*	0.22	19.74**	0.70	19.50**	0.76
LDH (U/L)	176.6	9.9	164.4	8.8	209.5	35.7	150.3	14.0	127.0	8.2
CPK (U/L)	1205.8	137.4	1036.8	198.4	923.0	126.6	553.3	93.1	458.0*	83.9

Values are expressed as mean ± SEM. Statistical significance was determined using the one-way ANOVA with Tukey’s posttest

*P < 0.05, **P < 0.01 compared with HCD

Vehicle; vehicle control, HCD; high-cholesterol diet control, CCGG-1X; high-cholesterol diet with 311 mg/kg/d of CCGG, CCGG-2X; high-cholesterol diet with 622 mg/kg/d of CCGG, CCGG-5X; high-cholesterol diet with 1555 mg/kg/d of CCGG

AST; aspartate aminotransferase, ALT; alanine transaminase, Alk-P; alkaline phosphatase, LDH; lactate dehydrogenase, CPK; creatine phosphokinase, BUN; blood urea nitrogen, UA; uric acid, TP; total protein

### Effects of CCGG supplementation on serum glycaemic markers and insulin resistance

**Fig. 4:**
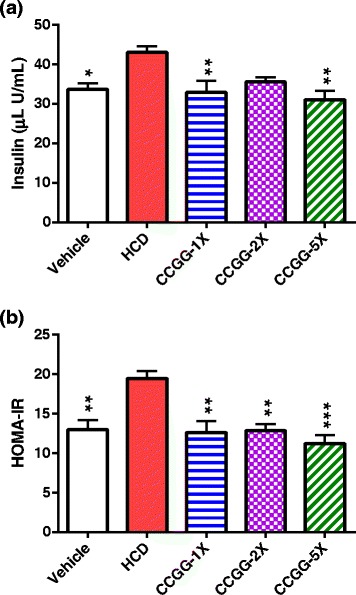
Effects of CCGG supplementation on glycemic markers. **a**; serum insulin levels. **b**; HOMA-IR index. Values are expressed as mean ± SEM. Statistical significance was determined using the one-way ANOVA with Tukey’s posttest. *P < 0.05, **P < 0.01, ***P < 0.001 compared with HCD. Vehicle; vehicle control, HCD; high-cholesterol diet control, CCGG-1X; high-cholesterol diet with 311 mg/kg/d of CCGG, CCGG-2X; high-cholesterol diet with 622 mg/kg/d of CCGG, CCGG-5X; high-cholesterol diet with 1555 mg/kg/d of CCGG

Glucose and insulin levels were measured after 6 weeks of CCGG treatment. A high-cholesterol diet increased glucose and insulin levels by 18.6 % (P = 0.21 vs. the vehicle group) and 27.7 % (P < 0.05 vs. the vehicle group), respectively. Insulin levels decreased in CCGG-supplemented groups by 17.4 %–27.7 %, compared with the HCD group (Fig. 4 (a)); however, glucose levels showed no significant change (Table 3). In addition, the HOMA-IR levels in the CCGG-supplemented groups were markedly improved by 33.8 %–42.3 %, compared with the HCD group (Fig. 4 (b)).

### Effects of CCGG supplementation on serum cytokine levels

**Fig. 5:**
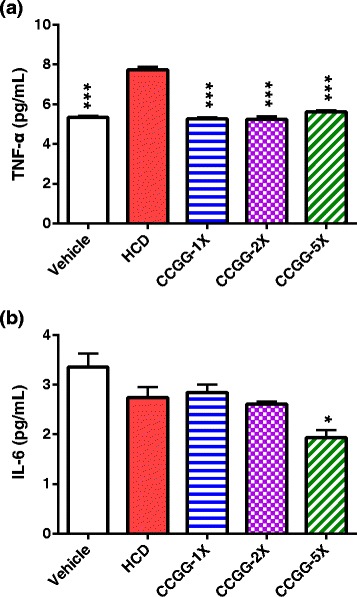
Effects of CCGG supplementation on pro-inflammatory cytokines. **a**; serum tumor necrosis factor-alpha levels. **b**; serum interleukin-6 levels. Values are expressed as mean ± SEM. Statistical significance was determined using the one-way ANOVA with Tukey’s posttest. *P < 0.05, ***P < 0.001 compared with HCD. Vehicle; vehicle control, HCD; high-cholesterol diet control, CCGG-1X; high-cholesterol diet with 311 mg/kg/d of CCGG, CCGG-2X; high-cholesterol diet with 622 mg/kg/d of CCGG, CCGG-5X; high-cholesterol diet with 1555 mg/kg/d of CCGG

Regarding serum proinflammatory cytokine levels, we determined an increase in TNF-α by 44.9 % in the HCD group, compared with the vehicle group (P < 0.001). However, no differences (P = 0.16) in IL-6 were observed between the vehicle and HCD groups (Fig. 5). Supplementation with CCGG reduced serum TNF-α by 27.4 %–32.3 % versus the HCD group (P < 0.001). In addition, a reduction of 29.6 % in serum IL-6 was observed in the CCGG-5X group, versus the HCD group (P < 0.05).

### Effects of CCGG supplementation on serum biochemical parameters

The safety of consuming CCGG was monitored at the end of the study. As shown in Table 3, no significant changes in total protein among the groups were observed compared with the HCD group. As indicators of hepatotoxicity, AST, ALT, Alk-P, albumin, total bilirubin and TP showed no differences among the groups versus the HCD group (P > 0.05). As indicators of nephrotoxicity, UA and creatinine showed no differences among the groups versus the HCD group (P > 0.05), whereas BUN was lower in the HCD group than in the four other groups. Similarly, as an indicator of cytotoxicity, LDH showed no differences among the groups versus the HCD group (P > 0.05). However, for CPK, an attenuation by 55.8 % in the CCGG-5X group, compared with the HCD group, was observed (P < 0.05).

### Effects of CCGG supplementation on hepatic and faecal lipid contents

Table 4 presents the results for hepatic and faecal lipid contents. In the liver, TG and cholesterol levels increased by 162.4 % and 547.2 %, respectively, in the HCD group compared with the vehicle group. CCGG supplementation significantly decreased TG levels (46.5 %, 46.0 % and 44.2 % in the CCGG-1X, CCGG-2X and CCGG-5X groups, respectively) and cholesterol levels (35.6 %, 50.2 % and 64.8 % in the CCGG-1X, CCGG-2X and CCGG-5X groups, respectively) compared with the HCD group. A dose-dependent effect was observed in hepatic cholesterol levels (r = -0.625, P < 0.001). In the faeces, TG, cholesterol and bile acid levels were increased by 27.5 %, 51.5 % and 83.2 % in the HCD group, compared with the vehicle group. However, no significant change was observed in the faecal TG in each of the groups, compared with the HCD group. Moreover, a decrease in faecal cholesterol was found in the CCGG-2X group, but not in the CCGG-1X or CCGG-5X groups, compared with the HCD group. Cholesterol tended to bind with bile and present as bile acid observed in the faeces. Supplementation increased the excretion of bile acid (37.1 %, 36.6 % and 30.1 % in the CCGG-1X, CCGG-2X and CCGG-5X groups, respectively), compared with the HCD group.

**Table 4:** Effects of CCGG supplementation on hepatic and faecal lipids

	Vehicle		HCD		CCGG-1X		CCGG-2X		CCGG-5X	
	Mean	SEM	Mean	SEM	Mean	SEM	Mean	SEM	Mean	SEM
Liver (mg/g)										
TG	142.3***	17.1	373.4	27.6	199.8***	21.6	201.8***	24.4	208.5***	23.0
Cholesterol	0.36***	0.02	2.33	0.38	1.50*	0.13	1.16**	0.12	0.82***	0.62
Faeces (mg/g)										
TG	21.8	2.1	27.8	2.2	37.6	3.8	37.3	1.9	17.8	2.1
Cholesterol	1.63***	0.05	2.47	0.09	2.38	0.70	1.97**	0.79	2.21	0.13
Bile acid	3.74**	0.26	6.85	0.46	9.39*	0.71	9.36*	0.32	8.91	0.67

Values are expressed as mean ± SEM. Statistical significance was determined using the one-way ANOVA with Tukey’s posttest

*P < 0.05, **P < 0.01, ***P < 0.001 compared with HCD

Vehicle; vehicle control, HCD; high-cholesterol diet control, CCGG-1X; high-cholesterol diet with 311 mg/kg/d of CCGG, CCGG-2X; high-cholesterol diet with 622 mg/kg/d of CCGG, CCGG-5X; high-cholesterol diet with 1555 mg/kg/d of CCGG

TG; triglycerides

### Effect of CCGG supplementation on the histopathology of tissues

**Fig. 6:**
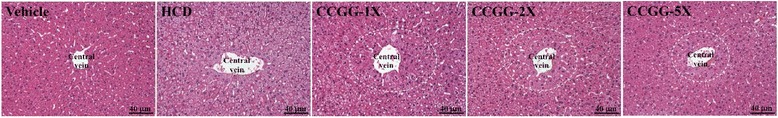
Histopathology of liver tissues. (H & E stain, magnification: 200×, Scale bar: 40 μm). Vehicle; vehicle control, HCD; high-cholesterol diet control, CCGG-1X; high-cholesterol diet with 311 mg/kg/d of CCGG, CCGG-2X; high-cholesterol diet with 622 mg/kg/d of CCGG, CCGG-5X; high-cholesterol diet with 1555 mg/kg/d of CCGG

The major organs, including the liver, epididymal fat, heart, kidney and lung, were examined. As shown in Fig. 6, histological analysis of the liver tissues revealed abundant foamy cells in the HCD group. In CCGG-supplemented groups, the inner area of the circle close to central vein presented foamy cells while the outer area presented relatively normal cells. CCGG brought inhibitory effects on formation of foamy cells extended from hepatic portal vein to central vein. In addition, the groups did not differ in histological observations of the epididymal fat, heart, kidney and lung tissues (see Additional file 1).

## Discussion

Dyslipidaemia is characterised by an increase in total cholesterol, low-density lipoprotein cholesterol, triglycerides or a combination of these abnormalities. The eventual clinical outcomes may include angina, myocardial infarction, arrhythmias, stroke, peripheral arterial disease, abdominal aortic aneurysm and sudden death [3]. Dyslipidaemia is one of the most severe metabolic diseases and a real problem currently encountered in the world. Instead of taking medicine, supplementation may be another approach to amelioration this chronic condition. In this study, we used golden Syrian hamsters fed a high-cholesterol diet (0.2 % cholesterol) and added an additional 10 % lard for our model. The animal model was shown to be appropriate because the major features of a lipid metabolism and atherosclerosis were similar between hamsters and human beings [24, 25]. This study was designed to investigate the beneficial effects of a CCGG supplement on lipid profiles, glycaemic markers, proinflammatory cytokines and related toxicity markers.

Regarding cocoa, Khan et al. [31] evaluated the effects of chronic cocoa consumption on the lipid profiles of volunteers exhibiting a high risk of cardiovascular disease. After 4 weeks, HDL-C levels were higher compared with TC, TG and LDL-C levels. In addition, the LDL/HDL-C ratio showed no significant change. However, Jia et al. [13] examined the short-term effect of cocoa product consumption on the lipid profiles of eight trials. LDL-C and TC levels were lowered, but no change in HDL-C was observed. No evidence for a dose-response effect was obtained. Regarding coffee, the effect of coffee on blood lipids remains controversial. Panchal et al. [32] revealed that coffee extract supplementation for 8 weeks could not change the abdominal obesity or dyslipidaemia in high-carbohydrate, high-fat rats. A meta-analysis conducted by Cai et al. [33] suggested that the serum levels of TC, LDL-C and TG may significantly increase in Western people who consume coffee daily. By contrast, a single-dose of coffee polyphenols could drastically modulate postprandial hyperlipidaemia in mice [34]. The effects of coffee beans are subsequently related to its phenol, oil and caffeine contents. Regarding green tea, the most effective polyphenol compounds against dyslipidaemia and other chronic diseases are known as catechins [10]. Particularly through modulating PPAR expression, green tea leaf extract was shown to improve lipid homeostasis by decreasing plasma and hepatic lipids and increasing serum adiponectin in rodent models [4, 5]. In addition, relatively few studies have focused on the benefits of Garcinia mangostana on blood lipids and have primarily investigated its antioxidant, antitumoural, antiinflammatory, antiallergy, antibacterial, antifungal and antiviral properties [11]. Xanthones (e.g. α-mangostin) are believed to be beneficial compounds. Nevertheless, Adiputro et al. [17] revealed a significant decrease in TC, LDL-C and TG as well as an increase in HDL-C in high-lipid fed rats after consuming mangosteen extract for 60 d.

We observed body weight and tissue weight. The liver, epididymal fat and heart were lower in the CCGG-5X group compared with the HCD group. These findings suggest that CCGG supplementation had potential effects on weight loss and organ protection. In addition, we observed that serum lipid levels were markedly increased in the HCD-fed hamsters compared with the normal chow-fed hamsters, and these increases were ablated by CCGG supplementation, even reversed to nearly baseline. Notably, simply dose-dependent reductions appeared in TC and LDL-C serum levels. Furthermore, hepatic lipid levels were increased in the HCD group. CCGG supplementation significantly decreased TG (by approximately 45 %) and cholesterol (dose-dependent) in the liver. In particular, hepatic steatosis improvement was observed in the histopathology. In addition, principally by the increase in the excretion of bile acid in faeces (by approximately 35 %), the absorption of a cholesterol diet was limited by CCGG supplementation.

Insulin resistance is a major underlying factor contributing to dyslipidaemia and hepatic steatosis [7]. HOMA-IR was reported to primarily indicate hepatic insulin resistance [35]. Improved HOMA-IR was revealed in previous studies after consuming 10-week flavan-3-ols-rich cocoa powder [12], 11-week 5-caffeoylquinic acid-rich coffee bean extract [19], and 8-weeks α-mangostin-rich mangosteen extract [36] in rodent models. However, the effect of green tea extract on insulin resistance remains controversial. Even increments in serum glucose and insulin levels were observed after 8-weeks EGCG-rich green tea extract intervention, leading to the development of diseases such as diabetes [37]. In our study, we determined that CCGG supplementation significantly decreased serum insulin levels and ameliorated the HOMA-IR index, but did not affect fasting blood glucose levels. Similarly, a lack of blood glucose regulation after treatment with phenolic compounds in mice, despite a significant reduction in insulin production, was reported [12]. It might take longer time either to induce or attenuate glucose level.

Inflammation leads to changes in lipid metabolism aimed at decreasing the harmful agents and promoting tissue repair involved in host defense [38]. As the adipocytes of individuals enlarge, the adipose tissue undergoes physiological alterations that subsequently affect systemic metabolism. First, macrophages accumulate within adipose tissue, leading to local inflammation. Several proinflammatory factors (including TNF-α and IL-6) are produced [1, 2]. Importantly, inflammatory cytokines, which are protagonists in plaque formation throughout the atherosclerotic vessel, are produced. Therefore, chronic inflammation is a major contributing factor to atherosclerotic disease. Regarding cocoa, a previous study [39] suggested that cocoa rich in flavonoids has the dose-dependent effects of diminishing TNF-α and MCP-1. Gu et al. [12] reported that a dietary proanthocyanidin-rich cocoa supplement decreased plasma IL-6 and MCP-1 instead of TNF-α. Regarding coffee, Yamauchi et al. investigated 5-weeks coffee and caffeine consumption in diabetic KK-Ay mice. Inflammatory markers of serum IL-6 were improved, but not monocyte chemoattractant protein-1 (MCP-1) [40]. However, a cross-sectional study revealed that coffee consumption does not affect serum TNF-α and IL-6 in Japanese workers [41]. Similarly, green tea has the potential effect of preventing inflammation by inhibiting MCP-1 [42] and TNF-α [43] expression. Regarding garcinia, α-mangostin plays a crucial role in antiinflammation. Both in vitro and in vivo studies have indicated the beneficial effects of suppressing the production of TNF-α [44, 45].

In our study, the results showed that serum TNF-α levels, but not IL-6, were increased in the HCD-fed hamsters. However, after the 6-weeks CCGG treatment, we observed that serum TNF-α levels were attenuated in three different-dosage-treated groups with a similar effect (approximately 30 % reduction). Furthermore, adiponectin is a secreted serum protein expressed exclusively in differentiated adipocytes. Adiponectin was shown to be highly correlated with metabolic syndrome and cardiovascular disease [46]. Adiponectin regulation is coordinated by other factors, including body mass index and serum TG [47]. A recent study [48] reported no change in serum adiponectin levels in 4-mo high-fat diet-induced obese rats. Thus, adiponectin levels might possibly be affected by some cooperative interaction because no differences occurred in serum adiponectin in our five groups. In addition, we observed that the CCGG supplement doses showed no sign of toxicity towards biochemical markers or histopathology and did not affect food or water intake.

On the whole, in CCGG, there were strong evidences that dyslipidaemia mediated by cocoa and green tea; insulin resistance mediated by cocoa, coffee and garcinia; inflammation mediated by cocoa, coffee, green tea and garcinia. Therefore, contributed by combined ingredients, we proposed that CCGG might exert a synergic effect. Accordingly, whether the combined ingredients indeed enhance the benefit and reduce the effective dose was crucial, since some studies [49–51] revealed their outcomes with disappointment after supplementation with multi-ingredient. However, our results are satisfactory. Based on conversion of animal doses to human equivalent doses from the US Food and Drug Administration, the doses of CCGG supplementation in hamster which are equivalent to 42, 84 and 210 mg/kg/d in human. Compared to single ingredient, less dose of CCGG supplementation might also be effective. Gu et al. [12] investigated the effect of cocoa powder supplementation on obesity-related inflammation in high fat fed obese mice. A 10-week 8 % unsweetened cocoa powder (approximately equivalent to 465 mg/kg/d in human) ameliorated obesity-related inflammation, insulin resistance and fatty liver disease. Panchal et al. [32] characterized the effects of coffee extract on a rat model of human metabolic syndrome. An 8-weeks 5 % aqueous coffee extract (approximately equivalent to 294 mg/kg/d in human) attenuated hypertension and impairment in glucose homeostasis without affecting abdominal fat deposition and plasma lipid profile. Luo et al. [4] investigated the effects of dietary supplementation with green tea on cholesterol and circulating immune complexes in SD rats fed with a high-cholesterol diet. A 30-d leaf powder of dose 1.0 or 2.0 g/kg body weight (approximately equivalent to 161 or 323 mg/kg/d in human) resulted in a significant decrease in plasma TC levels and circulating immune complexes and an increase in HDL-C. Adiputro et al. [17] evaluated the effect of mangosteen extract on lipid profile in rats fed a high lipid diet. A 60-d ethanolic extract of Garcinia mangostana pericarp at dosages of 400 or 800 mg/kg body weight (approximately equivalent to 65 or 129 mg/kg/d in human) significantly decreased cholesterol, TG and LDL-C levels. Concerning our results showed effective dose started at CCGG-1X group (equivalent to 42 mg/kg/d in human). Therefore, compared to previous studies focusing on single ingredient, the CCGG might have the synergic beneficial effects with relatively lower dose consumption. However, our limitation is that we cannot directly compare the effects of CCGG with each single ingredient. Additional studies are also required to assess the underlying mechanism that mediates the synergic effects of CCGG.

## Conclusion

In conclusion, we demonstrated hypolipidaemic effects of a 6-weeks dietary supplementation by using a combined extract (consisting of CCGG) in HCD-fed hamsters. CCGG markedly attenuated serum lipid profiles (TG, TC, and LDL-C) and hepatic lipid profiles (TG and cholesterol) with dose-dependent effects, and increased faecal bile acid content. Furthermore, HOMA-IR index and serum cytokine levels (IL-6 and TNF-α) involved in dyslipidaemia were improved, and no toxicity appeared. Thus, we propose that CCGG may have potential effects of ameliorating hyperlipidaemia, insulin resistance, liver steatosis and related inflammation.

## Additional file

Additional file 1:
**Histopathology of epididymal fat, kidney, heart and lung tissues.** a; epididymal fat tissue. b; kidney tissue. c; heart tissue. d; lung tissue. (H & E stain, magnification: 200×, Scale bar: 40 μm). Vehicle; vehicle control, HCD; high-cholesterol diet control, CCGG-1X; high-cholesterol diet with 311 mg/kg/d of CCGG, CCGG-2X; high-cholesterol diet with 622 mg/kg/d of CCGG, CCGG-5X; high-cholesterol diet with 1555 mg/kg/d of CCGG. (DOCX 3836 kb)
