# Supplementary material for: Effects of combined extract of cocoa, coffee, green tea and garcinia on lipid profiles, glycaemic markers and inflammatory responses in hamsters
Source: BMC Complement Altern Med. 2015 Aug 12;15:269. doi: 10.1186/s12906-015-0806-1 (PMC4533957; doi:10.1186/s12906-015-0806-1)
Supplement: Additional file 1: — Histopathology of epididymal fat, kidney, heart and lung tissues. a; epididymal fat tissue. b; kidney tissue. c; heart tissue. d; lung tissue. (H & E stain, magnification: 200×, Scale bar: 40 μm). Vehicle; vehicle control, HCD; high-cholesterol diet control, CCGG-1X; high-cholesterol diet with 311 mg/kg/d of CCGG, CCGG-2X; high-cholesterol diet with 622 mg/kg/d of CCGG, CCGG-5X; high-cholesterol diet with 1555 mg/kg/d of CCGG. (DOCX 3836 kb) [file 12906_2015_806_MOESM1_ESM.docx]

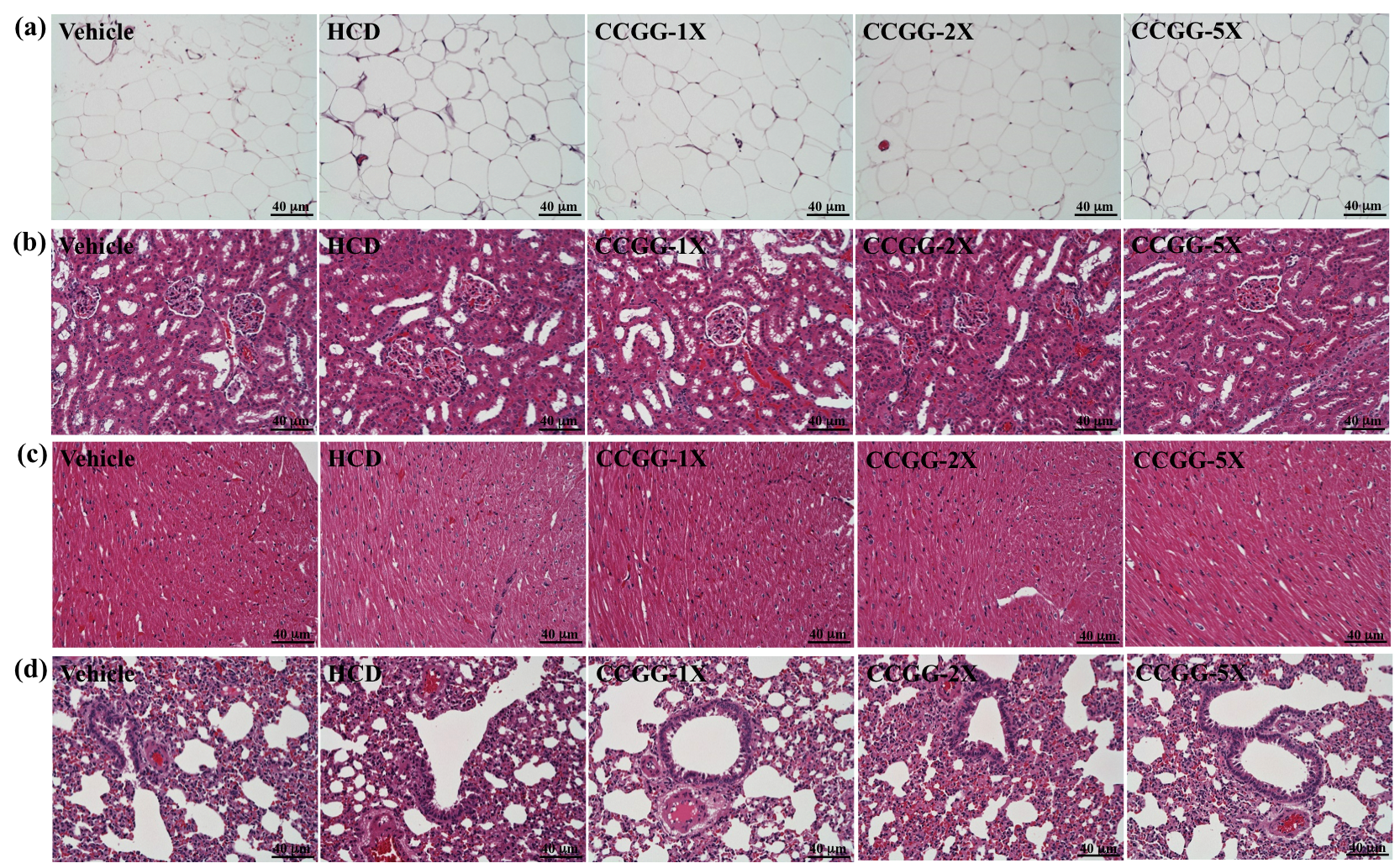


**Additional file 1. Histopathology of epididymal fat, kidney, heart and lung tissues**

a; epididymal fat tissue. b; kidney tissue. c; heart tissue. d; lung tissue.

(H & E stain, magnification: 200×, Scale bar: 40 μm).

Vehicle; vehicle control, HCD; high-cholesterol diet control, CCGG-1X; high-cholesterol diet with 311 mg/kg/d of CCGG, CCGG-2X; high-cholesterol diet with 622 mg/kg/d of CCGG;, CCGG-5X; high-cholesterol diet with 1555 mg/kg/d of CCGG
